# Mesoscopic physical removal of material using sliding nano-diamond contacts

**DOI:** 10.1038/s41598-018-21171-w

**Published:** 2018-02-14

**Authors:** Umberto Celano, Feng-Chun Hsia, Danielle Vanhaeren, Kristof Paredis, Torbjörn E. M. Nordling, Josephus G. Buijnsters, Thomas Hantschel, Wilfried Vandervorst

**Affiliations:** 1 IMEC, Kapeldreef 75, B-3001 Heverlee, Belgium; 2 Department of Mechanical Engineering, National Cheng Kung University, Tainan City, Taiwan; 3 Department of Precision and Microsystems Engineering, Delft University of Technology, Mekelweg 2, 2628 CD Delft, The Netherlands; 4 Department of Physics and Astronomy, KU Leuven, Celestijnenlaan 200D, B-3001 Leuven, Belgium

## Abstract

Wear mechanisms including fracture and plastic deformation at the nanoscale are central to understand sliding contacts. Recently, the combination of tip-induced material erosion with the sensing capability of secondary imaging modes of AFM, has enabled a slice-and-view tomographic technique named AFM tomography or Scalpel SPM. However, the elusive laws governing nanoscale wear and the large quantity of atoms involved in the tip-sample contact, require a dedicated mesoscale description to understand and model the tip-induced material removal. Here, we study nanosized sliding contacts made of diamond in the regime whereby thousands of nm^3^ are removed. We explore the fundamentals of high-pressure tip-induced material removal for various materials. Changes in the load force are systematically combined with AFM and SEM to increase the understanding and the process controllability. The nonlinear variation of the removal rate with the load force is interpreted as a combination of two contact regimes each dominating in a particular force range. By using the gradual transition between the two regimes, (1) the experimental rate of material eroded on each tip passage is modeled, (2) a controllable removal rate below 5 nm/scan for all the materials is demonstrated, thus opening to future development of 3D tomographic AFM.

## Introduction

Recent applications such as three-dimensional (3D) AFM tomography and tip-induced nano-manufacturing, are based on the capability of certain types of AFM tips to physically modify the scanned surface, e.g. remove material^1–5^. However, the exploitation of these techniques for a controlled removal of sample’s material requires the understanding of (nanosized) sliding contacts and their properties. Although the topic has been largely treated in the tribology community to interpret micro- and nanoscale wear mechanisms, the laws governing nanoscale wear remain elusive with various models available for the interpretation of the rich set of experimental observations. In addition, the large range of load forces and length scales experimentally accessible can complicate the interpretation of the wear data. A nanoscale model assuming thermally activated bond breaking has been introduced^6,7^. Jacobs and Carpick proposed a nanoscale wear mechanism based on atomic attrition resulting in an atom-by-atom removal of material^7^. Their experimental observation, using a Si tip sliding on a diamond surface, indicates a removal rate that depends exponentially on the stress that is interpreted by a chemical rate kinetics model^7^. On the other hand, at the mesoscale a model based on removal of clusters of material is generally accepted, which for example, can explain the formation of wear particles and residue composed of the removed material^8–11^. This has been supported by large-scale molecular dynamic simulations of diamond-like carbon (tips) sliding against a flat surface of the same material. In this case, the linear correlation between material loss and the normal load and sliding distance, was consistent with Archard’s law^11,12^. The latter states that the mass of material lost through wear is proportional to the normal load and the sliding distance multiplied by an empirical constant known as the wear coefficient (k). Although broadly applicable at the macroscale, Archard’s law and its underlying wear mechanisms are often not immediately extendable to the nanoscale. For example, a description of wear based on bond formation and rupture is often required in the case of nm-scaled asperities^6,13^. Due to the absence of a clearly defined edge for the contact area at this scale, the formation and rupture of interfacial bonds between the sliding contact and the sample induces the transfer of material. Additionally, the concept of multi-asperity contacts needs to be considered introducing a variable definition for the concept of sliding interfaces. Finally, the amount of wear will not only depend on the applied load force but also on the relative humidity^14^ and chemical reactivity of the active surfaces^15^. It goes without saying that understanding the mechanisms of wear and formulating a model for its control at the nanoscale is of great scientific and technological interest. A clear challenge is hereby the prediction of the parameters to control the tip-induced removal rate for various materials in order to satisfy a broad range of applications. Such predictive capacity would allow for example, to select the dedicated operational conditions of the tip for specific applications, i.e. a rapid material removal in case of nano-manufacturing or a gradual surface erosion in case of slice-and-view AFM tomography^16^. Here, we provide an extensive study about nanoscale wear as induced by sliding diamond nanocontacts with as prime objective to understand and improve the applications in the area of confined-volume characterization within semiconductor technology. First, we report on the usage of diamond tips to induce the removal of materials with varying hardness. The removal is studied as a function of the applied loads with as additional parameter the AFM raster scan conditions. In addition, the impact of the tip-sample interaction inside the worn regions is investigated in order to generate the fundamental understanding on the physical wear mechanisms. Based on these studies, we propose a model that combines Archard’s law with continuum mechanics contact-theory to interpret the tip-induced wear within a range of forces which is typically used in tip-induced material removal. Our model shows good agreement with the experimental data measured on a large set of materials such as metals, oxides and semiconductors.

## Sliding contact and rate of material removal

**Figure 1:**
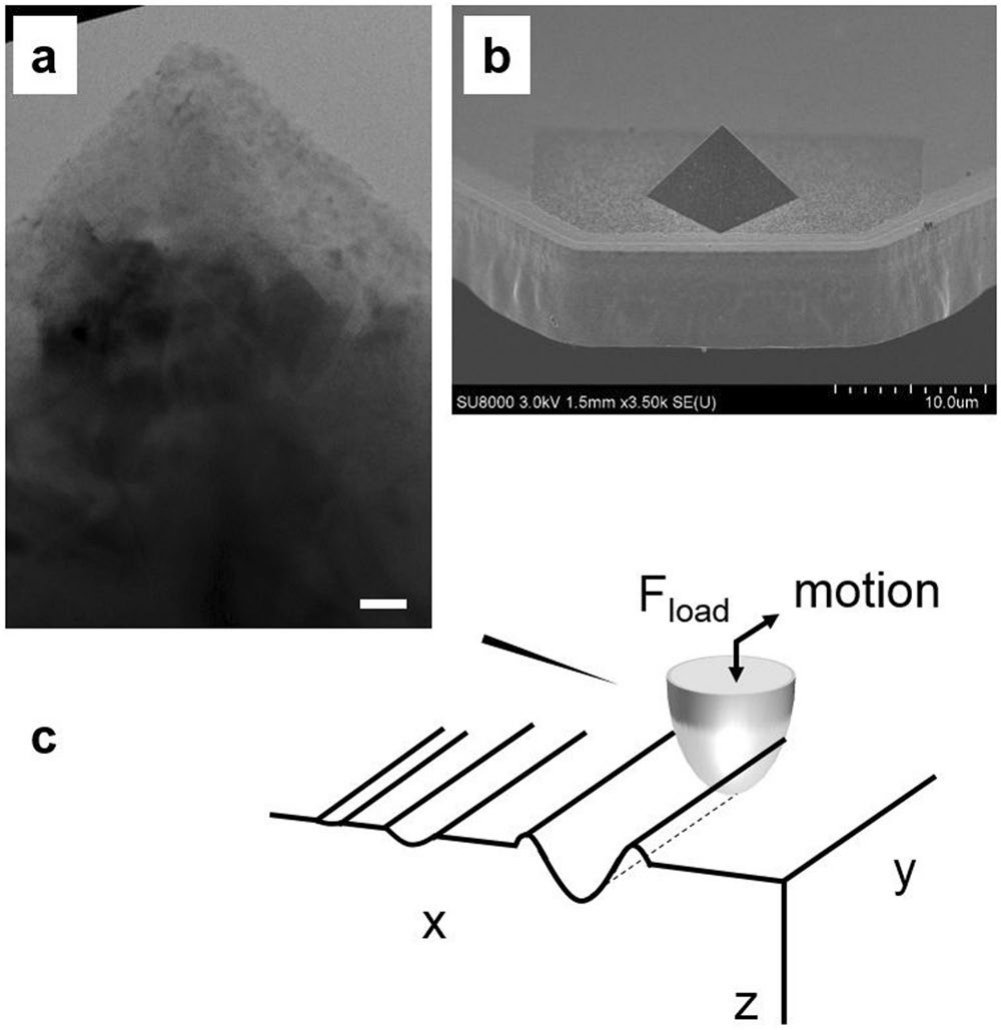
Details of the diamond tips used as sliding counterbody. (**a**) The apex of one of the diamond tips used in this work, as seen by high-resolution TEM (scale bar 20 nm). (**b**) The pyramidal shape of the tip is shown at lower magnification. (**c**) Schematic illustration of an AFM tip used to replicate a single-asperity sliding on a flat surface.

In our work we use exclusively B-doped diamond tips^17–19^ as the sliding counterbody in our experiments, their details as obtained with high resolution TEM, are shown in Fig. 1a,b. A schematic illustration of the tip replicating a single-asperity that slides on a flat surface is shown in Fig. 1c, more information on the quality of the diamond crystallinity is available here^10,20–22^. Depending on the selected materials and the stiffness of the cantilever, a mechanical removal of material occurs only when the tip overcomes a certain threshold force value. This value defines the transition between two different contact regimes: (1) at low forces, an elastic tip-sample contact regime characterized by a limited material removal, and (2), at high forces, plastic deformation regime in which trenches or grooves are mechanically induced on the surface^9,10,23,24^. This effect has been used through the years for nanoscale fabrication^25^ or layers removal^26^ for various (hard) materials such as metals and oxides, but also for (soft) polymers^27^. In general, when targeting substantial material removal during tip scanning, a tip load is selected such that one operates in the plastic deformation regime. Obviously, this force will depend on the material to be removed. In contrast, while doing Scalpel SPM we target a controlled removal of material on the nanometer scale, in order to collect 3D information of the sample with high depth resolution but nevertheless reasonable removal times. This requires the most precise control of the material removal which, as shown in this work, can only be achieved by operating at a load force at the boundary between the two contact regimes.

**Figure 2:**
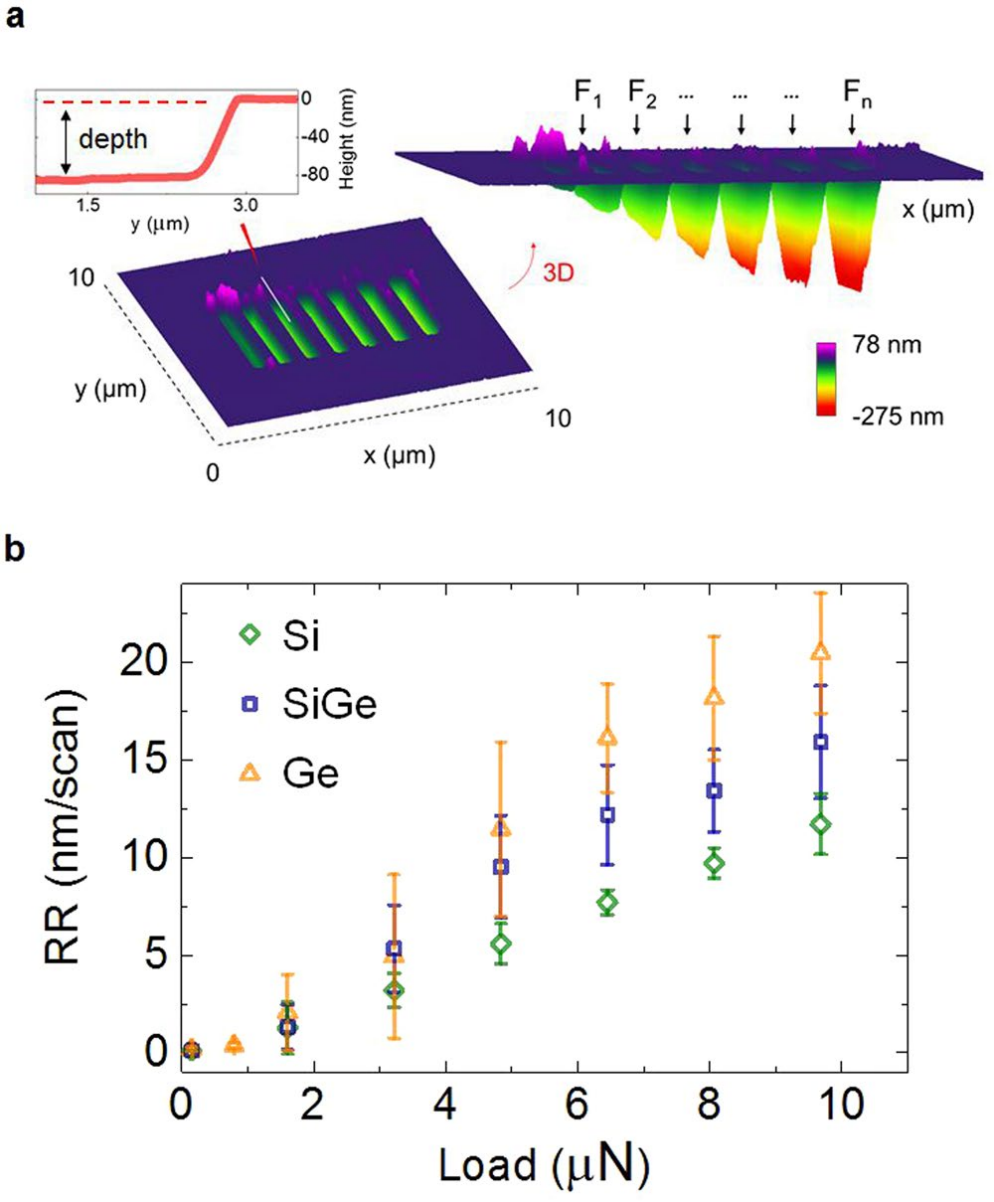
Experimental removal rate as a function of load forces. (**a**) Trench depths as seen by AFM topographical measurements across seven previously machined regions (size 4 µm × 500 nm) with increasing load force (F_1_ < F_2_… < F_n_). **(b**) Dependence of the removal rate on the tip load force for Si, SiGe and Ge for a diamond tip scanned in contact with the sample’s surface.

In order to study controlled material removal more quantitatively, the concept of tip-induced removal rate (RR) is introduced which defines the amount of material removed in each AFM scan. By one AFM scan we mean the process of scanning the AFM tip once over the entire area of interest with a process composed of multiple (overlapping) line scans. Figure 2 shows our experimental procedure to determine RR. For each experimental condition (e.g. load force), twenty consecutive AFM scans are performed in the same area (size 4 µm × 500 nm) leading to the formation of a (shallow) trench. Multiple trenches with increasing forces are formed leading to a series of worn areas as shown in Fig. 2a. Using the quantitative height measurement capabilities of AFM, the subsequent topographical AFM maps across these regions (seven in Fig. 2a) indicate the progressive increase in depth proportional with the tip load force. The RR is calculated by dividing the height difference inside and outside the trench (depth in Fig. 2a), by the number of scans. The same tip is used for one experimental run which contains trenches formed with forces ranging from 1 till 10 µN (which is the maximum load force accessible by our setup). For the sake of consistency, we use a constant scan size and tip velocity in all experiments, and we position the laser on the cantilever (approximately) on the same location, however as an accurate force estimate is not trivial the values of load force is also affected by a ± 20% dispersion induced by tip variations (as in Methods). Figure 2b shows the resulting RR as obtained at different load forces on three materials (Si, SiGe and Ge). Similar experiments are also performed on Pt, TiN (ceramic) and SiO_2_ to include cases of different hardness as shown in the Supplementary Information Figure S1. In Fig. 2b, each data point is the mean of five repeated experiments, each involving twenty consecutive AFM scans on the same area at constant load. One can immediately observe that in accordance with their reduced hardness, we observe a higher removal rate for Ge and SiGe as compared to Si (Fig. 2b). For all the materials, the RR shows a rapid rise when the tip force exceeds a threshold load force (F_th_ in range 1–4 µN). The error bars in Fig. 2b, indicate the variability linked to the use of different tips as this implies small variations in spring constant, tip radius and AFM laser positioning among various measurements (as demonstrated in Supplementary Information Figure S2). However, the results of Fig. 2b clearly indicate that for a given tip, the RR can be controlled from sub-nm to a few tens of nanometers by adjusting the load force. However, the weak dependency of the RR in the 1–4 µN force regime (Fig. 2b) may limit a precise sub-nm control of the tip-induced removal. As discussed in the following sections, the material removed is partially accumulating on the lateral and sliding edges of the scanned area and on the tip-body as shown in the Supporting Information Figure S3a.

**Figure 3:**
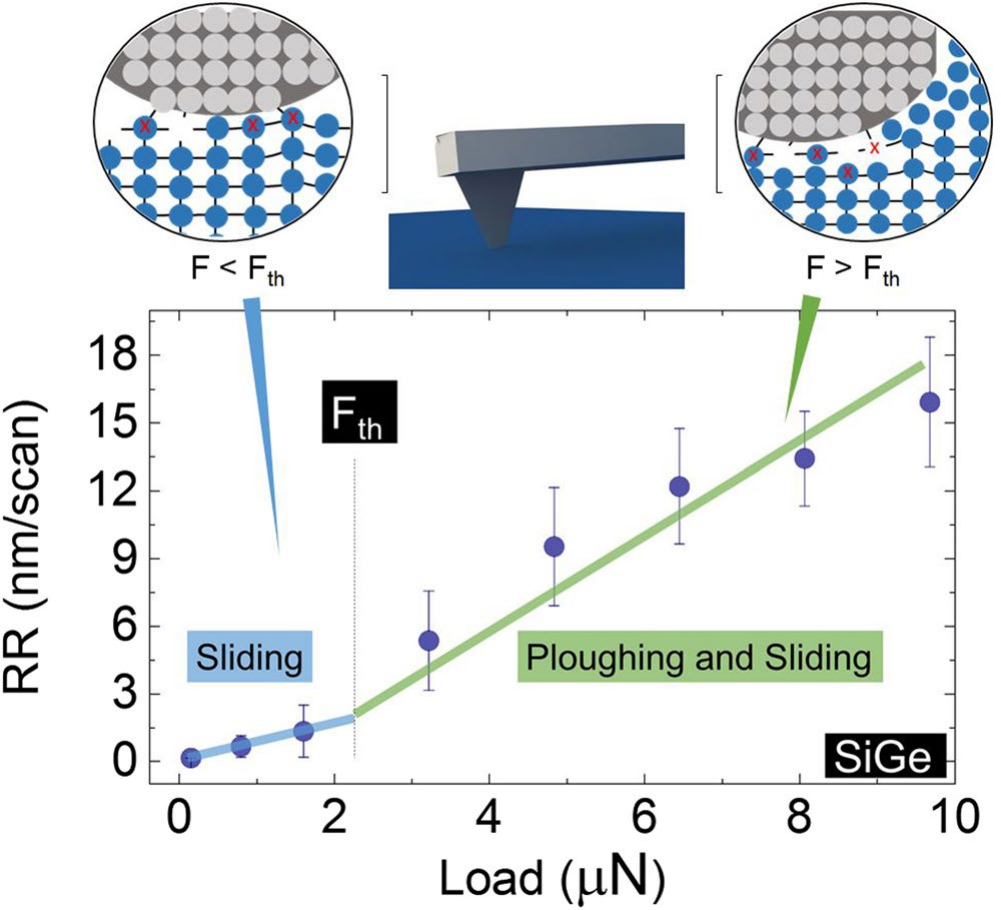
Two contact regimes are acting within the range of load forces investigated. The dependence of the removal rate on the tip load force for SiGe is shown with an added schematic representation of the two distinct contact mechanisms associated with different load forces.

The experimental values of RR in Fig. 2b, show a nonlinear dependence on the load force that appears when the contact pressure reaches the range of tens of GPa. This is visible in Fig. 2b in the 2–4 µN range, here the tip-sample contact-area is assumed to be ca. 10 nm^2^. The rapid growth of the RR over this threshold range can be interpreted as the existence of two contact regimes with distinctive physical mechanism governing the removal of material (Fig. 3). In Supporting Information Figure S6, we show for Ge that a two-mechanism model consisting of a linear increase with slope 0.15 before the threshold and a linear increase with slope 2.6 after the threshold provides a better explanation of the data than a single linear model with slope 2.2. The two-mechanism model stays within the region bounded by the smallest and largest observed value for each tested load and the root mean squared error (RMSE) is 5% smaller than the RMSE of the single linear model (2.8). Moreover, the two-mechanism model reflects the difference in the distribution of the residual errors below and above the threshold. Below the threshold the errors are not normally distributed because the RR is small and cannot be negative, while the errors agree well with a normal distribution above the threshold. Numerous mechanisms may contribute to this. For instance, with increasing load force the interaction will change from a sliding planar contact towards a pyramidal tip indenting the surface and thus plowing material. That in itself would already be enough to require models that scale with surface versus volume. Hence as the load force is increased, the contact regime progressively evolves from being dominated by the number of sliding contact-asperities (F < F_th_) to a full-plastic deformation^28^ of the sample material (F > F_th_) for the indented region within the area of contact. It is also important to recognize that with a contact area of ca. 10 nm^2^, it is relatively easy to reach a pressure as high as few GPa whereby the materials investigated show a brittle-to-ductile transition. The latter is very well known for semiconductors like silicon^29,30^, where this effect leads to the formation of a metallic β-Sn phase in the material which is used for scanning spreading resistance microscopy for example^30^. In other words, the rapid growth of the RR experimentally observed above F_th_ may be influenced by the brittle-to-ductile transition which is also material dependent. When considering a virtual separation between the two regimes (Fig. 3), a linear dependence between the material worn and the load force exists for both and the intercept of the two straights can be used to identify the value of F_th_. This allows one to apply the Archard’s law equation (Fig. 3) to fit the data. In our case, the application of Archard’s law provides two different wear coefficient values, one at low force (k = 10^−2^) and a second at high force (k = 10^−1^). Although these values can be considered in line with other observations respectively in ductile and brittle wear regimes^9^, they do not provide any real information about the intimate physical mechanisms of the removal. In addition, the range of experimentally observed wear coefficients covers several orders of magnitude (10^−7^–10^−1^)^31^, leading to a difficult generalization of this approach. Finally, a change of many orders of magnitude for the wear coefficient between the two contact regimes is not fully justified as the tip load force is not varying greatly in our experiment. Therefore, rather than study the trend in the RR curves, we probe directly the machined areas with different analysis techniques to collect information about the underlying wear mechanisms.

### Impact of the tip-induced material removal process

**Figure 4:**
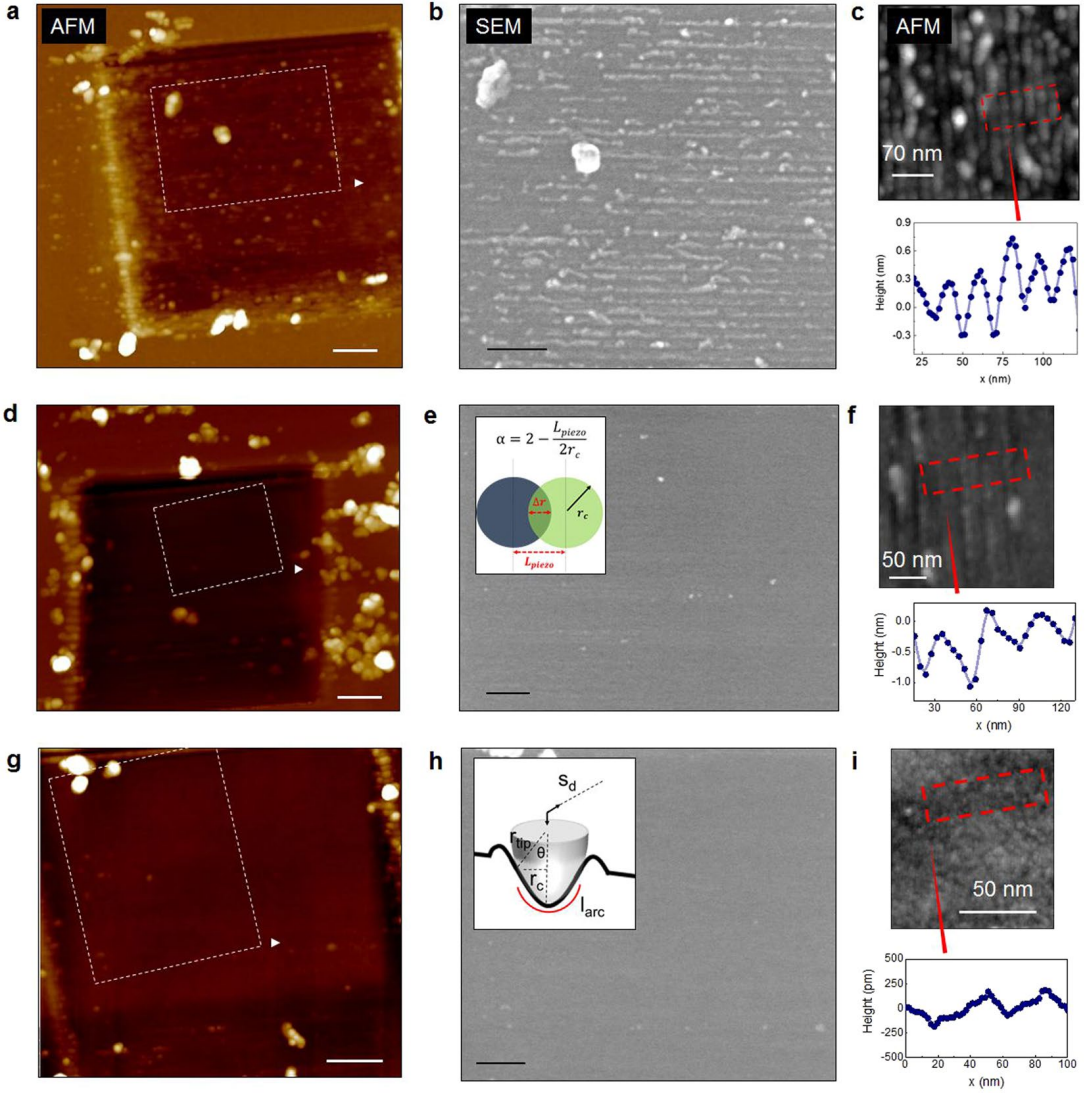
Impact of the material removal conditions on the surface quality of the machined area as studied by AFM and SEM. The same tip is used to scan three locations with the same load force and different line density (**a**) 128, (**d**) 256 and (**g**) 512 lines per image (scale bar 150 nm). The regions highlighted by a dashed box are imaged by SEM and shown in (**b**), (**e**) and (**h**), (scale bar 100 nm). 2D profiles are extracted using tapping mode AFM and are compared with the SEM images in (**c**), (**f**) and (**i**). Note, in the inset of (**e**) the geometrical considerations to calculate the overlap coefficient (α) are shown; while in the inset of (**h**) we show the details of the calculation for the deformed contact area.

To identify the role of the details of the material removal process we use Si as a model system, here tapping mode AFM and scanning electron microscopy (SEM) are used to assess the impact of the machining on the sample surface. Figure 4 shows the comparison between three different locations which are scanned at constant load force (5 µN) using the same number of scans, but with different line density i.e. 128, 256 and 512 lines per image. The characterization of the scanned area by tapping mode AFM is shown in Fig. 4a,d,g and that by SEM in Fig. 4b,e,h. Notably, Fig. 4 shows a clear dependence of the final surface quality on the density of lines used during the removal. The low line density leads to the formation of a rough corrugated surface with tip-induced debris that accumulates on the side of the grooves (Fig. 4a–c). On the contrary, a high line density improves the quality of the surface after the removal. This is visible for the case of 256 lines per image, and becomes even more pronounced at 512 lines as in Fig. 4g–i. In contrast with Fig. 4c–f, the final surface roughness of Fig. 4i reaches a value of 0.28 nm (measured over 1 × 1 µm^2^) which represents a relatively low degradation of the machined area compared to the value 0.13 nm measured outside the machined area. The debris accumulation inside the area is highly reduced with the material now almost entirely clustering on the side of the scanned area (Fig. 4g–i). Interestingly, also the depth of the worn area has a clear dependence with the line density, leading to a removal depth of ca. 2 (128 lines), 2.5 (256 lines) and 3 nm (512 lines) respectively in Fig. 4a,d,g. It is clear that both parameters, surface quality and removal efficiency, are influenced by the line density, or specifically by the ratio between the distance of two adjacent AFM lines and the tip-sample contact area (inset Fig. 4e). In essence, when a scan size and lines density/scan are selected, the space between two consecutive lines (L_piezo_) is imposed by the electronics of the AFM system that actuates the piezoelectric element moving the tip. Depending on the tip-sample contact radius (r_c_), a portion of space Δr exists between two consecutive line scans which has already be machined (Δr = 2r_c_ − L_piezo_ for L_piezo_ < 2r_c_ and Δr = 0 for L_piezo_ > 2r_c_). It goes without saying that the higher the line density, the smaller the L_piezo_ and therefore the larger Δr will be. To include this in any removal model, we introduce a contact overlap coefficient (α = 2 − (L_piezo_/2r_c_)) as a parameter that accounts for the number of times that the sliding contact passes over the same area. The overlap has a dramatic impact on the final surface quality, because scanning with a high line density leads to a cleaner finishing of the surface with less residues and a smoother profile of the induced grooves. At the same time, a large contact overlap can be considered as a multiple passage on the same location during the AFM scan, thus increasing the effective RR, as experimentally observed.

Interestingly, the periodicity of the grooves induced as in Fig. 4c,f clearly indicates the appearance of interference effects due to the value of line density and apparent tip radius. Although the rigorous treatment of these interference patterns is out of the scope of the present work, it is worth to mention that they appear similar to the surface patterns visible in Laser-induced breakdown spectroscopy (LIBS)^32,33^. However, here the observed patterns are not determined by the laser wavelength but rather to the fact that L_piezo_ is not an integer multiple of r_c_. Finally, it is worth noting that when considering the role of line density at reduced load force (F < F_th_), the creation of characteristic lines (due to the ploughing effect) disappears (not shown) and the surface roughness is maintained similar to the case of Fig. 4i. In view of this, the formation of the surface patterns appears to be linked to the onset of the plastic deformation regime and as such the threshold force can be considered as the onset of the plastic deformation regime.

### Tip-induced material removal: interpretation and modeling

**Figure 5:**
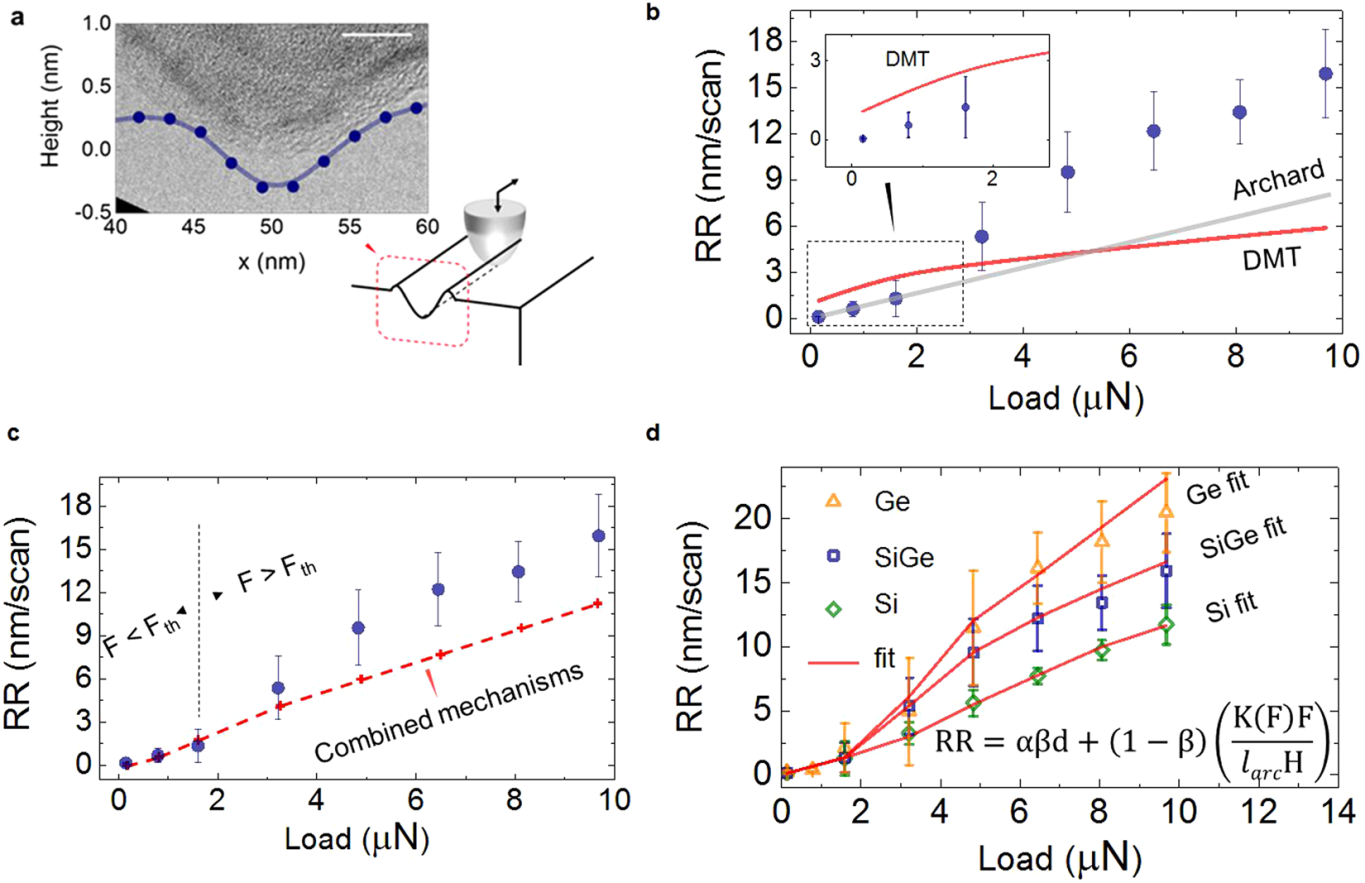
Modeling of the experimental RR. (**a**) Very good agreement is observed between the observed groove and the diamond tip, as shown by the over imposition of a 2D profile with a high resolution TEM image of the tip apex, scale bar 5 nm. (**b**) The RR of SiGe is plotted with the addition of the indentation depth as calculated with the DMT model and the Archard’s law equation derived by fitting the low-force regime. (**c**) In our model we propose to sum of the two contributions weighted by a coefficient (β) which is used to define the onset of the elastic-to-plastic transition and a coefficient (α) to account for the overlap between adjacent lines. This combination provides an improved approximation of our model with the experimental RR, however a certain underestimation of the high-force regime remains. (**d**) A complete version of our model requires also a pressure-dependent wear coefficient K(F) which provides a good fit to all our experimental RR in Si, SiGe and Ge.

From the previous observations, we can now attempt to provide a comprehensive model covering the entire force regime. For that purpose one can start from two observations i.e. at low force the material removal (Fig. 3) occurs in the absence of any tip-induced ploughing. In this case, the removal can be considered to occur in the ‘sliding regime’, and is based on a nanoscale wear mechanism involving bond formation and rupture similar to the atom-by-atom removal^7,13^. In this regime Archard’s law may be applied implying that due to the small contact area and low stress induced by the small force, a long sliding distance is required to remove nanometers of materials within the region of interest. Whereas this represents a region for ultra-precise removal, its efficiency is very small (high removal time required). On the contrary, at higher load forces (larger than F_th_ in Fig. 3) the clear appearance of debris (outside the scanned area as well as on the tip apex), and tip-induced ploughing topography indicates the combination of two processes. First, a tip-induced plastic deformation and second, a transport of atoms in front of the tip with partial accumulation on the leading edge of the tip-apex as well. Indeed our observations reveal that the shear traction causes the accumulation of material at the leading edge of the sliding contact and its accumulation and detachment around the scanned area (Supporting Information Figure S3). By comparing the AFM with the SEM images, it is possible to extract the 2D (AFM) profiles of the stressed areas as shown in Fig. 4c,f,i. Here, the ploughing effect induced by the tip is clearly visible, particularly for the case of low line density (Fig. 4c) where each individual passage of the tip can be identified (note, in this case L_piezo_ = 15.6 nm). The average indentation depth of the aforementioned grooves ranges from 0.3–0.5 nm, while the assumption of 10 nm^2^ tip-sample contact area seems very well supported by the experimental observations (Fig. 5a). Therefore, while modeling the experimental RR, we now consider a combination of tip-induced plastic deformation and sliding. For the deformation within the area of contact, we consider the problem of a static indenter and calculate the indentation depth. To simplify the complex problem of the elastic–plastic deformation, we consider the static indentation of our tip with the sample and calculate the indentation depth using the Derjaguin, Muller and Toporov (DMT) model which predicts a deformed contact profile equivalent to the Hertz theory and accounts in addition for the adhesion in elastic contacts^28^. The DMT model is accurate in case of stiff materials, small sphere radii and weak long-range adhesion force in line with our experimental setup. The expression for the contact radius becomes

1 $${r}_{c}={[\frac{3}{4}\ast {r}_{tip}\ast ((\frac{1{-{\rm{v}}}_{1}^{2}}{{E}_{1}})+(\frac{1{-{\rm{v}}}_{2}^{2}}{{E}_{2}})\ast (F+4\pi \gamma {r}_{tip}))]}^{1/3}$$

where r_tip_ is the nominal tip radius, E_1_ is the Young’s modulus and ν_1_ is the Poisson ratio of the boron doped diamond tip and E_2_ and ν_2_ is the same for the surface material, F is the loading force, and γ is the work of adhesion. The values of the coefficients that we use in our calculations are reported in Table S1. From the value of r_c_, we can estimate the static indentation depth using the value of the tip radius (nominally r_tip = _15 nm). Using the small-angle approximation, θ = sin^−1^(r_c_/r_tip_), l_arc_ can be approximated by l_arc_ = 2 * r_tip_ * θ^34^. Here θ is the angle insisting on the r_c_ segment as shown in the inset of Fig. 4h. The height of the deformed contact, i.e. the indentation depth^34^, will provide the static contribution due to plastic deformation induced by the tip acting as a single asperity object:

2 $$d={r}_{tip}-({r}_{c}\ast sin\theta )=\frac{{{r}_{c}}^{2}}{{r}_{tip}}$$

In a very simplistic model (ignoring the effect of line overlap) one would expect to find a direct correlation between the calculated tip-induced indentation depths and the experimental RR. Indeed, Pearson’s linear correlation coefficient of the indentation depth versus RR is 0.94 for Ge, but linear regression between RR and the indentation depth reveals a poorer fit than RR and the load force (RMSE 2.8 vs. 2.2 and adjusted R^2^ 0.89 vs. 0.94) as in Supporting Information Figure S7. Although both show an increase with the applied force their growth profile is different. The calculated results of the tip-induced indentation depth are shown in Fig. 5b (red line) and over imposed to the experimental RR data (SiGe). The indentation depth predicted by the DMT model exceeds the material removed experimentally at low-force and falls behind the RR in the high-force regime. This is due to the fact that the tip-induced material removal is not just a static-indentation but it is the combined result of tip-induced indentation, plastic deformation and trailing. Based on these observations, we can now start to build a more comprehensive model for RR. In the low force regime we can use simply Archard’s law whereby one needs to relate the sliding distance to AFM scanning distance.

3 $$V=K(F)Fx/H$$

where V is the volume of the removed material, K is the wear coefficient, F is the applied load force, x is the sliding distance (the number of scan lines * scan length), H is the hardness of the material under test. Based on previous observations the wear coefficient (K) is considered dependent on the contact area and friction force^11^. In practice, this means that we allow it to vary with the load force. Since we probe the removal rate, that is the removed volume at the end of a single scan, (eq. 3) is transformed into RR = V/A_scan_ where A_scan =_ l_arc_ * x. The latter is obtained considering l_arc_ as the length of the arc section insisting over the angle θ which is formed between the tip and the diameter of the contact area (as illustrated in Fig. 4h), multiplied by the sliding distance. Note, that since Archard’s law only depends on the sliding distance, the parameter α does not need to be included. Using this equation we find in the low-force range (i.e. 0–3 µN in Fig. 3) a wear coefficient in the range of 10^−2^ consistent with previous work on similar materials^9,23,24^.

As a second removal mechanism, in the high force regime we assume that the plastic deformation (indentation) dominates, with however a modification to account for the line overlap. Hence in this case

4 $$RR=d\ast \alpha $$

Where d describes the plastic deformation per scan induced by the tip acting as an indenting single asperity. The contact overlap coefficient (α) is used to account for the number of times that the tip passes on the same area. Alpha is an important parameter because as shown in Fig. 4 it can dramatically affect the morphology of the removed area and the overall RR. In practice the two mechanisms are acting together while the tip contact is dragged on the surface and the resulting model is combined of two terms:

5 $$RR=\beta d\alpha +(1-\beta )\ast (K(F)F/({l}_{arc}H))$$

In eq. (5) we introduced also β as a weighing factor probing the proximity of the elastic-to-plastic transition, we define β = ξ/(ξ + 1) whereby ξ = F/F_th_ such that β ranges between zero and one over the entire load range. In essence, by doing this we can define a range of forces (material-dependent) for the elastic-to-plastic transition once the tip’s spring constant is known. Finally, as refinement to eq. (3) and consistent with previous observations the wear coefficient (K) is considered as dependent on the contact area and friction force^6^. Therefore, we allowed in eqs (3–5) this term to be variable with the load force. Obviously its variation needs to be limited to magnitude consistent with its physical nature. As such we have limited its possible fluctuation at 20x the original value, measured in the low force regime (where we assume no plastic deformation). It can be easily seen that without this modification i.e. the fixed wear coefficient (Fig. 5c), the model shows the correct trend although underestimates the experimental values in the high force regime. On the contrary, Fig. 5d shows the fitting results of the model in eq. 5 (red lines) for different materials. We observe a good agreement between the experimental RR values and our model. The fitting shown in Fig. 5d is repeated in Supporting Information Figure S4 for more materials i.e. SiO_2_, Pt and TiN.

### Tip wear and material re-deposition

**Figure 6:**
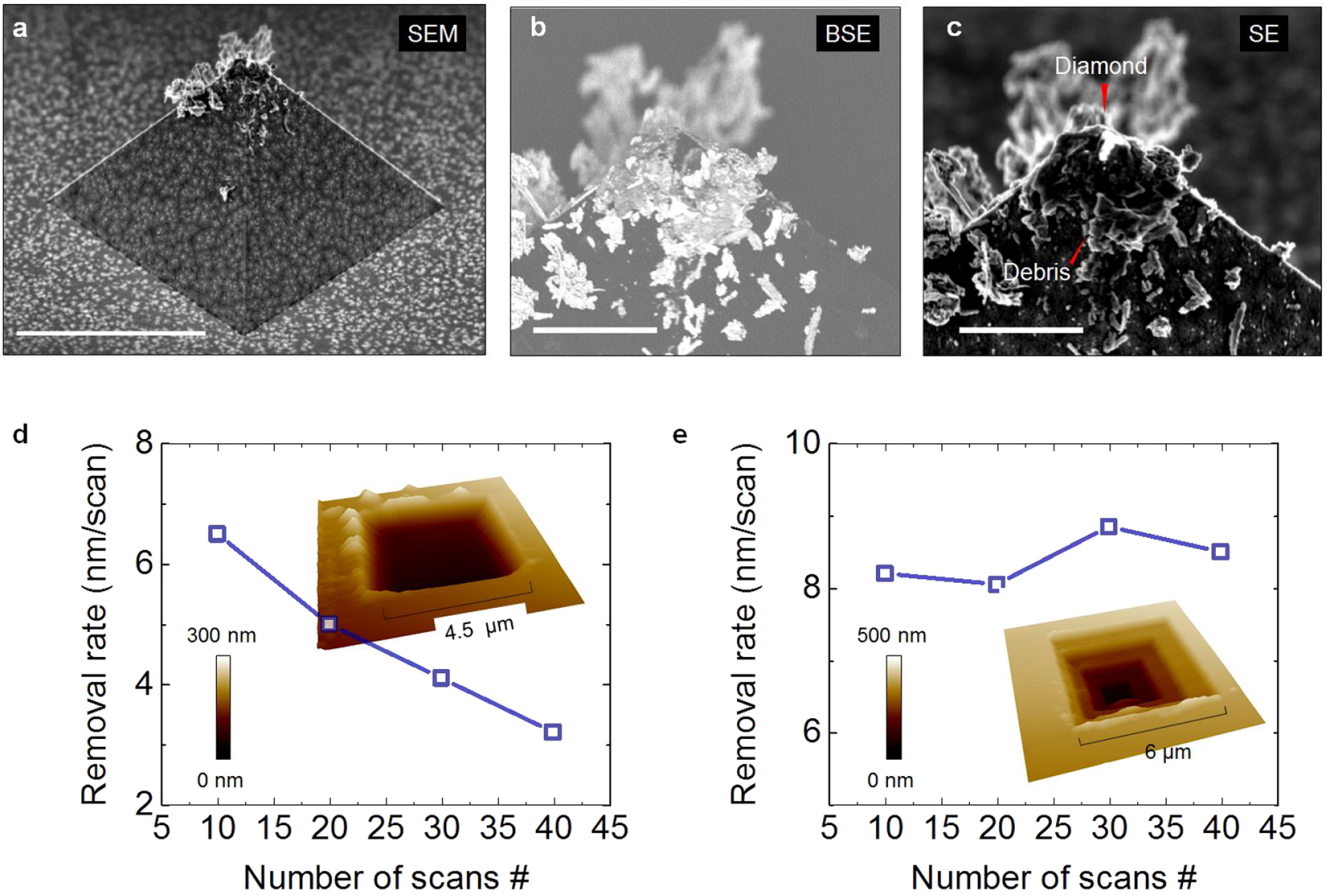
Impact of the tip-apex contamination and material re-deposition. (**a**) SEM image of the diamond tip after usage (scale bar 5 µm). (**b**) Zoom-in image by SEM back-scattered electrons (scale bars 1 µm) and (**c**) secondary electrons. Thanks to the high secondary-electron emission for diamond, the area of the tip-apex used in contact with the sample is clearly revealed. (**d**) Apparent decrease in the RR induced by the re-deposition of material in the worn area. (**e**) This artifact can be removed by a progressive reduction of the scan area during the removal which guarantees a more stable RR.

Finally, we discuss the impact of the removal on the tip’s mechanical integrity and the consequences of the material accumulation as a source of undesired effects in our method. Indeed, despite the high wear resistance of diamond, it is legitimate to consider the impact of an intense material removal on the tip apex itself too. In particular, tip blunting and its potential impact on the RR needs to be considered. This can be done by a direct inspection of the tip-apex by SEM after prolonged material removal (Fig. 6a). Here, the formation of clusters of material debris on the tip body and their accumulation on the edges of the tip are clearly visible. Figure 6b,c shows the details of the tip-apex. Thanks to the different electrons emission property of diamond compared to other materials (i.e. its low-vacuum-barrier height), a strong contrast is obtained discriminating clean diamond area from the accumulated material debris (Fig. 6c). When we assume that the portion of the tip-apex which is not covered by residues, was in direct contact with the sample, this result shows that despite the large amount of removed material, the diamond tip-apex can still be used as a sliding contact. Unfortunately, the SEM inspection carried out only after the sliding experiment, does not yield enough information to assess the effect of the potential tip blunting on the RR. As discussed in the previous sections, the RR is strongly affected by the tip-sample contact area and therefore also by the tip-apex. Hence one expects that when the load force is kept constant, the RR stays constant over time, provided that the geometry of the tip-apex does not change during the scan. Therefore, some dedicated experiments aimed at monitoring the RR over the number of scans can indirectly be used to study potential changes in the tip-apex. This is presented in Fig. 6d,e. A decreasing trend in the RR as function of repeated scans (same force and scan size) is shown in Fig. 6d. One can consider this effect as the result of the tip-apex becoming blunter leading to a reduced RR. However, this interpretation ignores the fact that with increasing scan number, the scanned area becomes rather deep (>200 nm for 40 scans) such that the material accumulating in front of the tip can no longer be deposited at the edges of the scanned area but rather stays inside the trench. This effect reduces the effective RR as the tip is now scanning over the previously removed material, and the result is a false decrease in the RR as in Fig. 6d. The latter represents an artifact introduced by the scanning conditions, i.e. the re-deposition of the removed material inside the trench. This can easily be demonstrated, as we show in Fig. 6e. Here, the same methodology is used, however this time the scan size is progressively reduced over cycles (inset Fig. 6e), so that we limit the re-deposition of the removed material inside the area under investigation (inset Fig. 6d). Excluding the cases of severe tip-apex ruptures or contaminations, repeated experiments such as these indicate a limited impact of the material removal on the physical changes of the tip-apex and thus the RR. This effect, is also not surprising in view of the constant material transport at the trailing end occurring at the edge of the sliding contact. While a shallow trench will allow the removed material to accumulate on the side of the scan area and the tip’s sides, at increasing depths the material starts to pile up on the sidewalls of the trench and progressively is re-deposited and smeared on the scanned area. Our study proves that this effect is not caused by a change in the tip geometry because the apparent reduction in the RR can be immediately recovered when the tip is moved in a new area and a new removal is performed (Supporting Information Figure S5). Hence, our results indicate that a major showstopper for a constant RR (and ultra-precise control of the removal rate) is the progressive re-deposition of materials when scanning at a fixed scan size. We observe that this has a major effect on the effective removal capability of the tips. It is worth mentioning that in view of the high precision required by our removal, the range of load-forces is also relatively small which combined with the hardness of the diamond crystallites constituting the apex explain the limited impact on the tip integrity.

In summary, we have demonstrated that the mechanisms of mesoscale material removal established for a sliding AFM tip in contact with the sample surface, can be described by the combination of tip-induced indentation, plastic deformation and material trailing. Based on the experimental evidences observed by AFM and SEM in the machined areas, we could introduce a model that provides an accurate fit for the experimental removal rate (RR) in different materials such as metals, oxides and semiconductors. Although the non-linear RR at the transition between an elastic and a plastic surface deformation imposes some limitation for the accurate prediction of the RR, we could demonstrate (for all materials) a sub-3 nm RR which can be applied to further develop AFM-based tomographic techniques. In addition, our work clearly indicates the major role of the scan line density on RR, as well as on the quality of the treated surface, both impacting on the final 3D information.

## Methods

The layers investigated in this work are blanket samples grown on a Si substrate, TiN, Pt and SiO_2_ are deposited by PVD while Ge is grown by CVD, finally, the SiGe is in composition Si_0.30_Ge_0.70_. SPM measurements, including non-contact tapping mode AFM were performed in air on a Digital Instrument, Dimension 3100 equipped with a controller Nanoscope version 3a. All the measurements are performed using in-house developed full-diamond tips (FDT) with a cantilever of spring constant 10 N/m, these tips are commercially available with the name of Pyramidal Solid Diamond Probes. Inferred by the geometrical parameters, the dispersion of the specified spring constant can be affected by fluctuations on the values of the cantilever, the main one is certainly represented by the cantilever thickness which has a ± 20% possible variation, in line with most of the commercially available tips. The scan frequency (determining the scan speed) in all the experiments is 0.6 Hz. The deflection sensitivity of the tip under study was measured before, during and at the end of the removal process to assess a possible unrecoverable bending of the cantilever. The machined areas and the contamination of the tip apex were inspected by scanning electron microscopy (SEM) with a SU8000 (Hitachi, Japan) system.

## Electronic supplementary material

Supplementary information
