# Supplementary material for: Mesoscopic physical removal of material using sliding nano-diamond contacts
Source: Sci Rep. 2018 Feb 14;8:2994. doi: 10.1038/s41598-018-21171-w (PMC5813091; doi:10.1038/s41598-018-21171-w)
Supplement: Supplementary file 1 — Supplementary information [file 41598_2018_21171_MOESM1_ESM.pdf]

Supplementary Information

Mesoscopic physical removal of material using nano-diamond sliding contacts

Umberto Celano[1*], Feng-Chun Hsia[1,2], Danielle Vanhaeren[1], Kristof Paredis[1], Torbjörn E. M. Nordling[2], Josephus G. Buijnsters[3], Thomas Hantschel[1], Wilfried Vandervorst[1,4]

[1] IMEC, Kapeldreef 75, B-3001 Heverlee, Belgium

[2] Department of Mechanical Engineering, National Cheng Kung University, Taiwan

[3] Department of Precision and Microsystems Engineering, Delft University of Technology, Mekelweg 2, Delft, The Netherlands

[4] KU Leuven, Department of Physics and Astronomy, Celestijnenlaan 200D, B-3001 Leuven, Belgium

*corresponding author: umberto.celano@imec.be

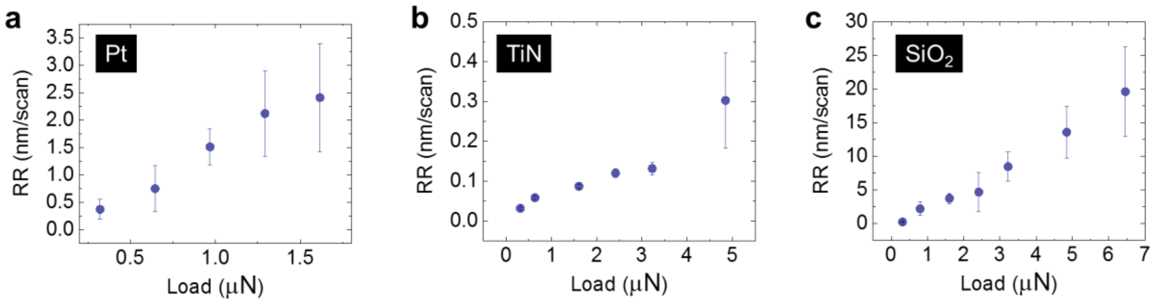

Figure S1 Experimental observation of the dependence of the removal rate on the tip load force for Pt, TiN and SiO_2 (thickness 60, 30 and 300 nm respectively). As for the main text the values are experimentally measured by a diamond tip scanned in contact with the sample's surface. Due to their respective hardness, each material shows a different RR, which is as expected higher for Pt compared to TiN. In particular TiN shows sub-nm removal for the entire set of accessible load forces. The latter is consistent with previous observations where the removal of 30 nm TiN required tens of hours.[1] It is worth noticing that the SiO_2 shows here a

relatively high RR, which can be explained by the quality of the oxide which is this case is a porous sputtered dielectric.

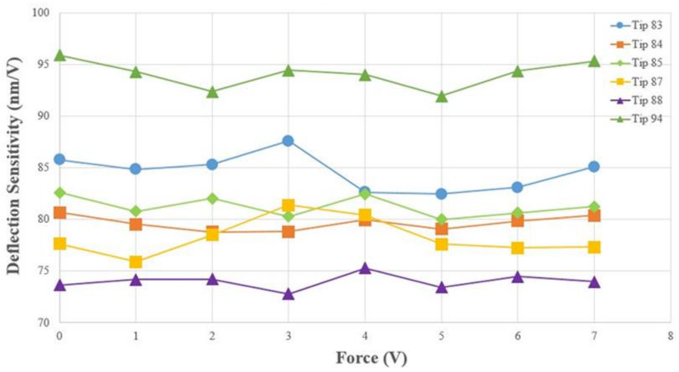

Figure S2 (a) To correlate a change in the RR with a structural change of the tip cantilever, we repeatedly perform force-curve spectroscopy during the material removal. From the force curves we extract the deflection sensitivity of our tips (nm/V). As this number is determined by the mechanical properties of the cantilever, this can be used to check the cantilever sanity during removal. The latter is shown in the figure, where despite their relative spread around a nominal value of 80 nm/V, our tips do not show a degradation for the deflection sensitivity even after removal at relatively high forces. The latter is extracted after 10 scans which are repeated as in figure at different load forces. No visible degradation occurs during this stress. Note, this does not mean that the tip apex is not damaged but that the cantilever as a mechanical spring system is not changed.

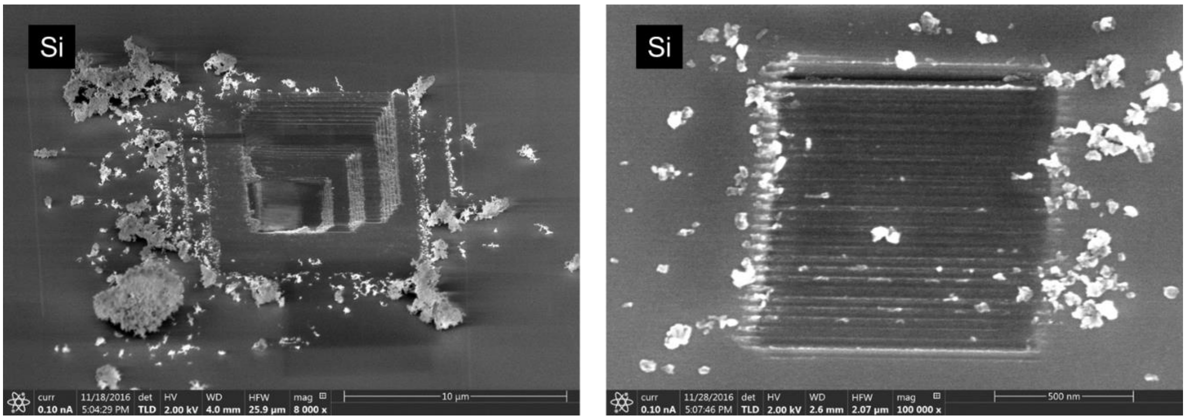

Figure S3 The trenches induced by our tip during the material removal are imaged by SEM for the case of low line density (128 lines per scan). (a) In this image, the progressive reduction of the scan size is visible, with clusters of removed materials which accumulate on the side of the scanned areas. (b) Similarly, we report the case of a scan with fixed dimensions (2 x 2μm^2) for which the same effect is observed. Note, here also the presence of the scanning grooves described in the main body of this paper.

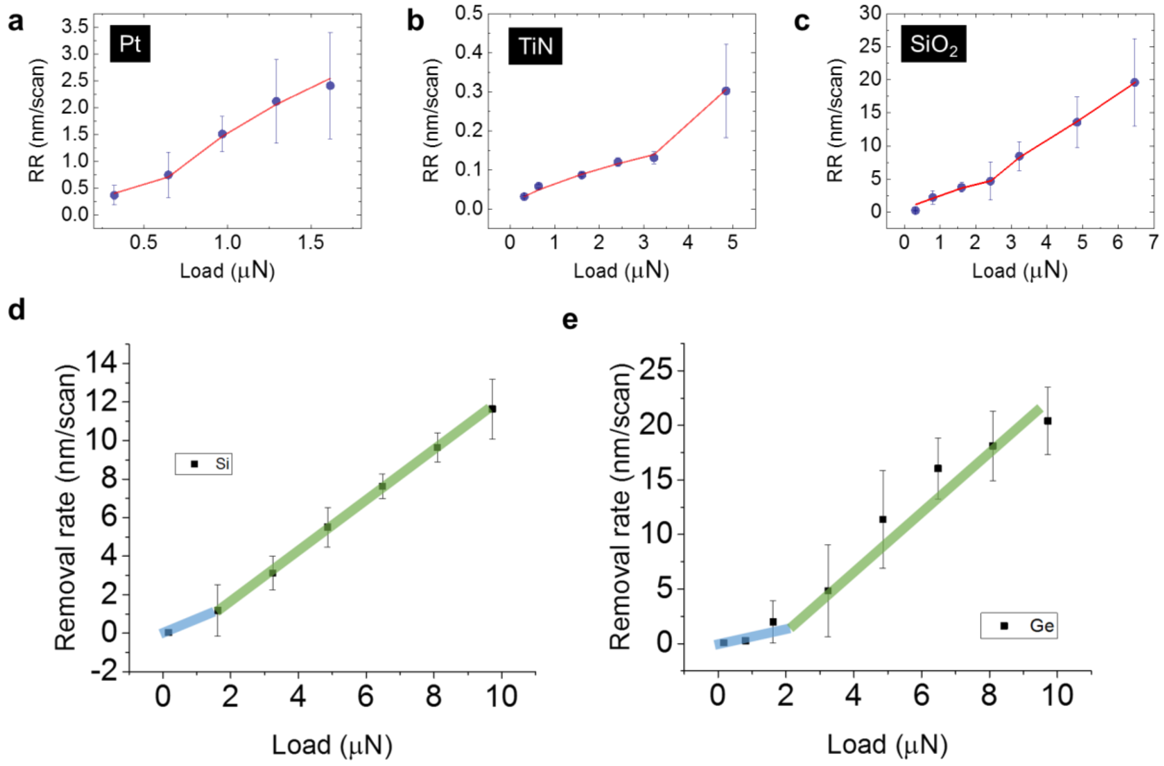

Figure S4 Fitting of the experimental RR based on our model (eq. 5) for the case of (a) sputtered SiO_2, (b) Pt and (c) TiN. Note, that Pt is the only material among these that shows the same behavior observed in the main text for the semiconductors. The latter is due to its relatively low hardness compared to SiO_2 and TiN. Particularly, these two materials have a higher hardness shifting their threshold force in a range not always reachable by our configuration. As result, their RR follows a more linear behavior with the tip-induced

ploughing occurring at higher forces. (d, e) The individual RR vs. Force curves for the Si and Ge dataset reported in the main text Figure 2 is shown.

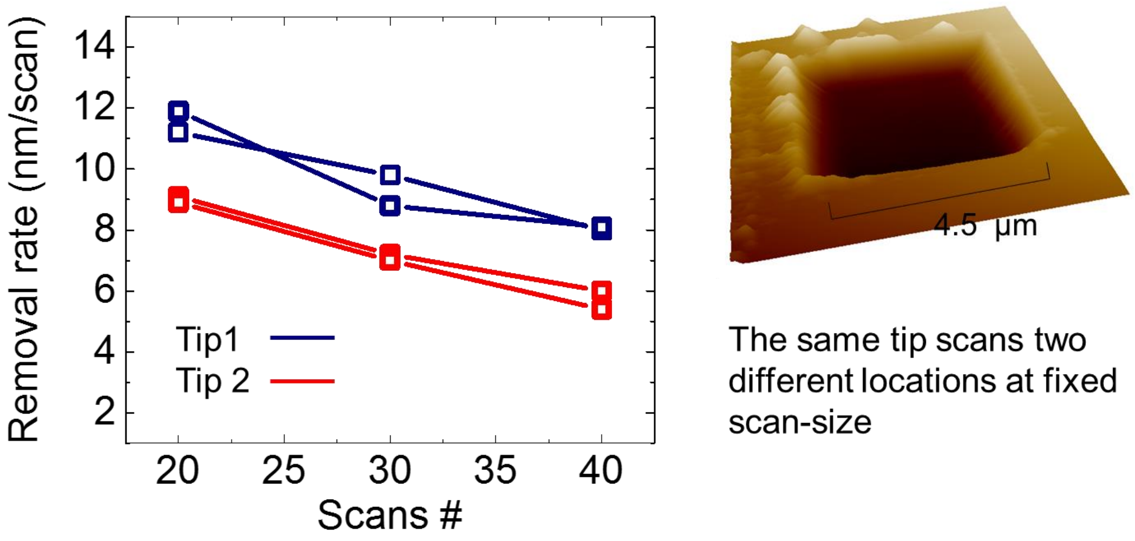

Figure S5 The observed decay in the RR as function of prolonged scans can be related to the re-deposition of material and not to a change in the tip-apex sharpness. The latter is shown in figure where two tips, are scanned for 40 cycles in different area while the RR is recorded. For both tips the decay in the RR is a recoverable effect, and as such is not related to a physical modification in the tip-apex structure. Indeed for both tips after the first 40 scans are performed and the RR is reduced, the same RR can be restored by simply moving the tip to a new surface location, obtaining a similar decay for the RR as the scan size is kept fixed during the material removal.

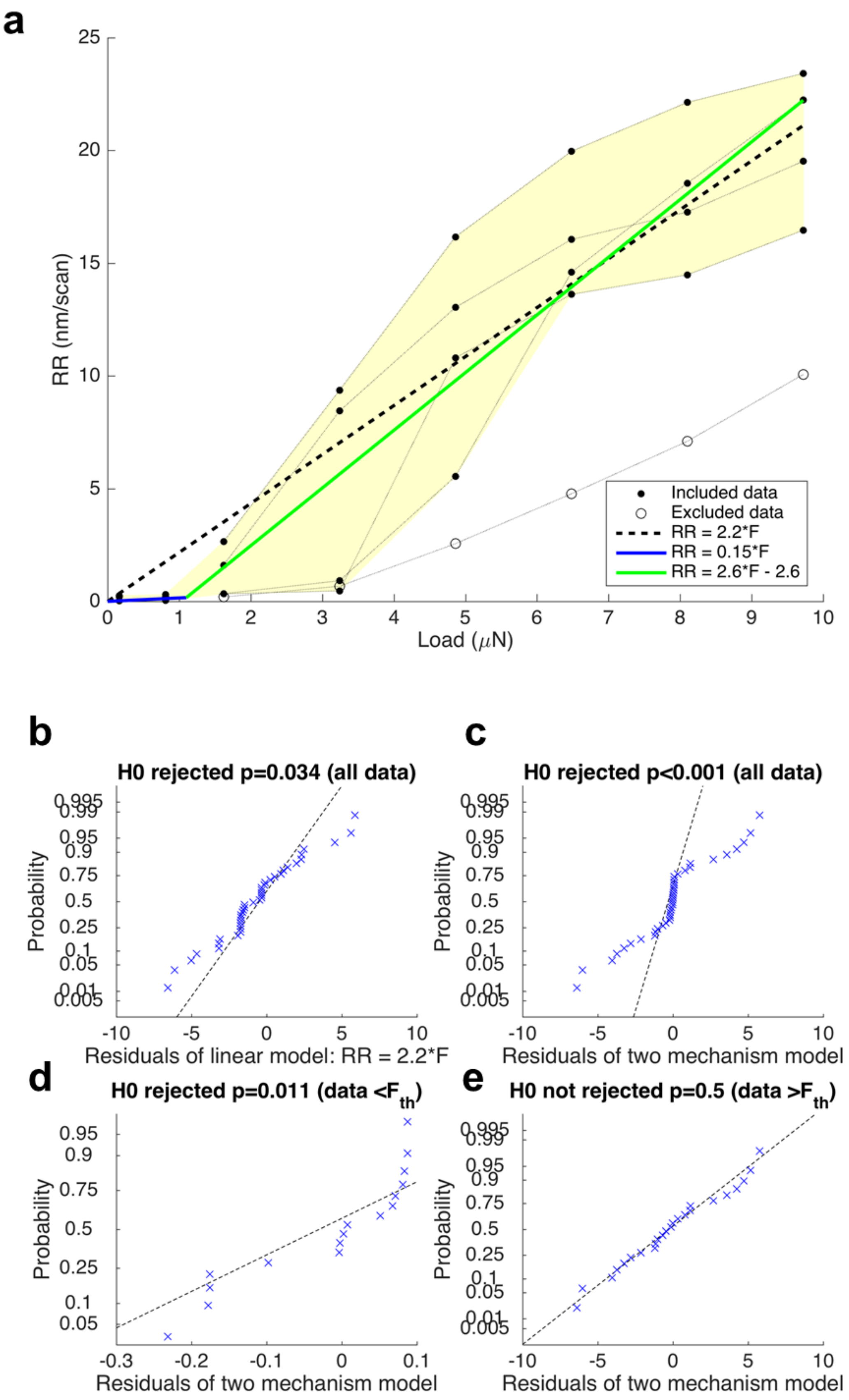
a
RR (nm/scan)
Load (μN)
Included data
Excluded data
RR = 2.2*F
RR = 0.15*F
RR = 2.6*F - 2.6
b
H0 rejected p=0.034 (all data)
Probability
Residuals of linear model: RR = 2.2*F
c
H0 rejected p<0.001 (all data)
Probability
Residuals of two mechanism model
d
H0 rejected p=0.011 (data <F_th)
Probability
Residuals of two mechanism model
e
H0 not rejected p=0.5 (data >F_th)
Probability
Residuals of two mechanism model

Figure S6 Regression analysis of the Ge dataset reveals that a two mechanism model consisting of a linear increase with slope 0.15 before the threshold (1.1 μN) and a linear increase with slope 2.6 after the threshold provides a better explanation of the data than a single linear model with slope 2.2. The two mechanism model stays within the region bounded by the smallest and largest observed value for each tested load (yellow shading in a) and the RMSE (2.6) is 5% smaller than the RMSE of the single linear model (2.8). The assumption that the residual is normally distributed in all data points is rejected for both models (b,c). Below the threshold the errors are not normally distributed because the RR is small and cannot be negative (d), while the errors agree well with a normal distribution above the threshold (e).

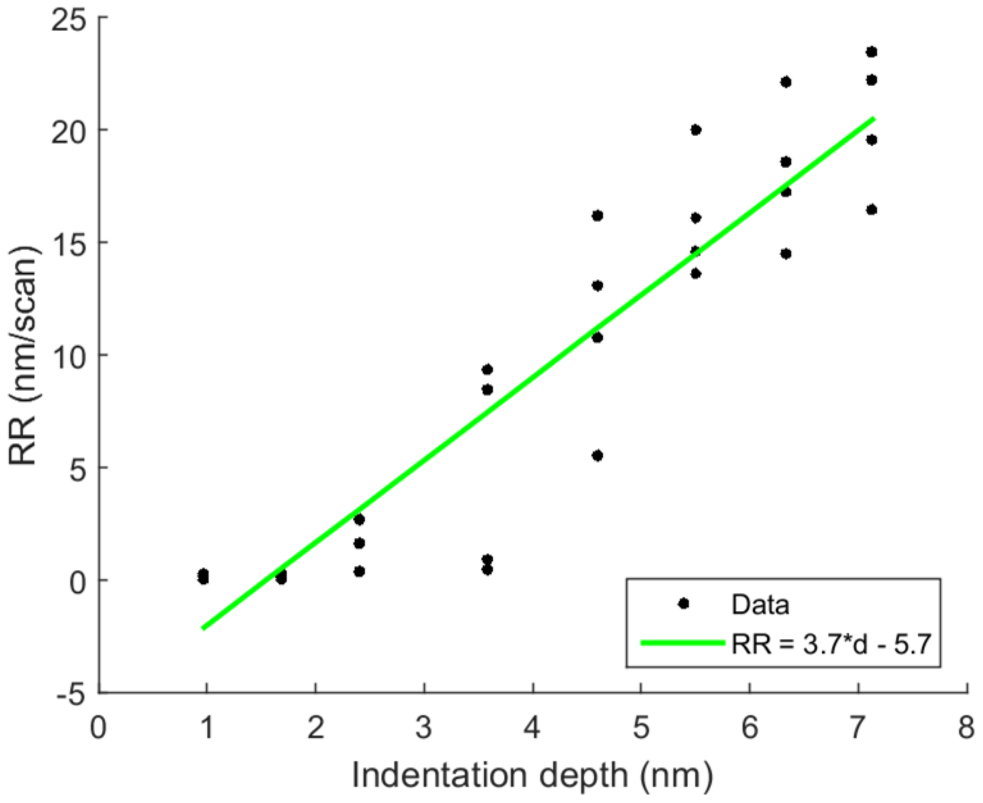

Figure S7 Removal rate versus the indentation depth (d) of Ge shows a clear correlation as expected. Pearson's linear correlation coefficient is 0.94, but linear regression between RR and

the indentation depth reveals a poorer fit than linear regression between RR and the load force (RMSE 2.8 vs. 2.2 and adjusted R2 0.89 vs. 0.94).

Table S1: Mechanical properties and surface energy of materials.

**Material**	**Young's Modulus (GPa)**	**Poisson's ratio**	**Hardness (GPa)**	**Surface Energy ($\frac{J}{m^2}$)**
Diamond	1220 [1], [2]	0.2 [1], [2]		
Silicon	130 [3]	0.064 [3]	13 [4]	2.13 [5]
$Silicon_{0.3}$ $Germanium_{0.7}$	110.5 [6]	0.27	10[4]	2
Germanium	102.7 [7]	0.28 [7]	10[4]	1.84 [5]
Silicon Oxide	75 [8]	0.17 [9]	18 [10]	0.054 [11]
Platinum	168 [12]	0.38 [13]	0.55 [14]	2.34 [15]
Titanium Nitride	650 [16]	0.25 [17]	20 [16]	0.063 [18]

References

[1] K. E.Spear andJ. P.Dismukes, *Synthetic diamond: emerging CVD science and technology*, vol. 25. John Wiley & Sons, 1994.

[2] B.Banerjee, *An introduction to metamaterials and waves in composites*. CRC Press, 2011.

[3] M. aHopcroft, W. D.Nix, andT. W.Kenny, "What is the Young's Modulus of Silicon?," *J. Microelectromechanical Syst.*, vol. 19, no. 2, pp. 229–238, Apr.2010.

[4] L. J.Vandeperre, F.Giuliani, S. J.Lloyd, andW. J.Clegg, "The hardness of silicon and germanium," *Acta Mater.*, vol. 55, no. 18, pp. 6307–6315, Oct.2007.

[5] R. J.Jaccodine, "Surface Energy of Germanium and Silicon," *J. Electrochem. Soc.*, vol. 110, no. 6, p. 524, 1963.

[6] J. J.Wortman andR. A.Evans, "Young's Modulus, Shear Modulus, and Poisson's Ratio in Silicon and Germanium," *J. Appl. Phys.*, vol. 36, no. 1, pp. 153–156, Jan.1965.

[7] J. J.Wortman andR. A.Evans, "Young's Modulus, Shear Modulus, and Poisson's Ratio in Silicon and Germanium," *J. Appl. Phys.*, vol. 36, no. 1, pp. 153–156, Jan.1965.

[8] C.-A.Jong, T.-S.Chin, andW.Fang, "Residual stress and thermal expansion behavior of TaOxNy films by the micro-cantilever method," *Thin Solid Films*, vol. 401, no. 1–2, pp. 291–297, Dec.2001.

[9] M. T.Kim, "Influence of substrates on the elastic reaction of films for the microindentation tests," *Thin Solid Films*, vol. 283, no. 1–2, pp. 12–16, 1996.

[10] B.Bhushan, O. E.Scholar, andC.Microtribology, "Nanotribology and nanomechanics of MEMS devises," *IEEE Micro Electro Mech. Syst. Work.*, pp. 91–98, 1996.

[11] B.Pignataro, G.Grasso, L.Renna, andG.Marletta, "Adhesion properties on nanometric scale of silicon oxide and silicon nitride surfaces modified by 1-octadecene," *Surf. Interface Anal.*, vol. 33, no. 2, pp. 54–58, 2002.

[12] R.Martinez-Duarte, P.Renaud, andM. J.Madou, "A novel approach to dielectrophoresis using carbon electrodes," *Electrophoresis*, vol. 32, no. 17, pp. 2385–2392, 2011.

[13] M.Topfer andH.Knake, "High Temperature Mechanical Properties of the Platinum Group Metals," *Platin. Met. Rev.*, vol. 45, no. 2, pp. 74–82, 2001.

[14] B. Y. N.Loginov, A.V.Yermakov, L. G.Grohovskaya, andG. I.Studenok, "Annealing Characteristics and Strain Resistance of 99.93 wt.% Platinum," *Platin. Met. Rev.*, vol. 51, no. 4, pp. 178–184, Oct.2007.

[15] W. H.Lee, K. R.Vanloon, V.Petrova, J. B.Woodhouse, C. M.Loxton, andR. I.Masel, "The equilibrium shape and surface energy anisotropy of clean platinum," *J. Catal.*, vol. 126, no. 2, pp. 658–670, 1990.

[16] E.Bailey, N. M. T.Ray, A. L.Hector, P.Crozier, W. T.Petuskey, andP. F.McMillan, "Mechanical Properties of Titanium Nitride Nanocomposites Produced by Chemical Precursor Synthesis Followed by High-P,T Treatment," *Materials (Basel).*, vol. 4, no. 12, pp. 1747–1762, Oct.2011.

[17] R. O. E.Vijgen andJ. H.Dautzenberg, "Mechanical measurement of the residual stress in thin PVD films," *Thin Solid Films*, vol. 270, no. 1–2, pp. 264–269, Dec.1995.

[18] C.-C.Sun, S.-C.Lee, W.-C.Hwang, J.-S.Hwang, I.-T.Tang, andY.-S.Fu, "Surface Free Energy of Alloy Nitride Coatings Deposited Using Closed Field Unbalanced Magnetron Sputter Ion Plating," *Mater. Trans.*, vol. 47, no. 10, pp. 2533–2539, 2006.
